# Understanding Internal Medicine Residents' Rheumatology Knowledge Base

**DOI:** 10.7759/cureus.60689

**Published:** 2024-05-20

**Authors:** Irvind Buttar, Nana Jinjolava

**Affiliations:** 1 Internal Medicine, Lenox Hill Hospital, New York, USA; 2 Rheumatology, Lenox Hill Hospital, New York, USA

**Keywords:** hospital survey, internal medicine residency, american college of rheumatology (acr), academic rheumatology, hospitalist resident education quality improvement

## Abstract

Rheumatology is one of the internal medicine subspecialties that residents train to become proficient in during their internal medicine training. Our study sought to understand how residents across all postgraduate year levels felt in terms of comfortability and exposure to rheumatology. We focused on the subjective measurement of resident knowledge and exposure rather than objective data. A five-question survey was distributed to all 75 residents of Lenox Hill Hospital's Internal Medicine categorical residency program, from PGY1 to PGY3. When asked if they get enough exposure or feel confident treating patients with rheumatology diseases, 96% of residents responded no. When asked about their confidence in boards, the average response was a 3/10. The residents at our program voiced a strong concern for lack of exposure and education. Other studies and institutions have shown this to be a problem that has also been seen with poor test performance on the subject. We explore educational modalities to help improve this gap in education.

## Introduction

Rheumatology is one of the internal medicine subspecialties that residents train to become proficient in during their internal medicine training. While reviewing previous studies, ​​Garneau showed that there has been a lack of exposure and confidence in the field for internal medicine residents. This then leads to internists being uncomfortable with treating these conditions [1]. Katz et al. showed that internal medicine residents had less confidence in their rheumatology skills when compared to other subspecialties. Not only do residents have poor confidence in their rheumatology skill set but their attendings also believe their proficiency is low [2]. While these studies and others we use to help understand our data were conducted at large university hospitals, such as the University of Chicago and Duke University, we aimed to understand the confidence and comfortability of our residents at a community hospital in Manhattan. At this hospital, there is no rheumatology fellowship or required rotation, so residents gain their exposure through their inpatient and outpatient internal medicine rotations. The subject is learned about through patient cases and didactics and direct exposure with rheumatology attendings. 

While the importance of treating these conditions is important, the parameters of a successful medical education have undeniably been based on test performance. The American Board of Internal Medicine’s blueprint for the exam shows that rheumatology is 9% of the exam material tested. This is the same percentage as endocrinology and gastroenterology. Garneau has shown that residents are much more comfortable with those subspecialties over rheumatology [3]. Leverenz used training exams to evaluate residents' knowledge of certain topics and performance which reflected the gaps in knowledge were also objective [1]. Medical education has been transforming over the past decade, and while teaching styles vary, the repeated lack of confidence in this subject has remained consistent [4]. 

Our study sought to understand how the residents across all postgraduate year levels felt in terms of comfortability and exposure to rheumatology. Previous institutions with rheumatology fellowship programs have shown that residents did not have confidence in their skill set [5]. We went to evaluate it in our setting where a fellowship program was not present. We identify our residents' confidence levels and topics that they feel they are exposed to through a survey [6,7]. 

## Materials and methods

Lenox Hill Hospital's Internal Medicine program has 75 categorical residents ranging from PGY1 3. The hospital is a community hospital in New York City, with affiliations to Northwell Zucker School of Medicine. There are no rheumatology fellows in-house as there is no fellowship, but there are rheumatology attendings as a part of the internal medicine department. We internally reviewed different approaches to education that are used within our institution and outside our institution prior to sending out the survey to prevent bias when considering different approaches that may be used based on the results. 

We wanted to understand the perception the residents had of their understanding and knowledge of the subject. To obtain this subjective data, we sent out a six-question survey made on Microsoft Forms titled “Rheumatology Exposure Assessment.” The survey contained a mix of questions that used yes/no responses, the Likert scale (1-10, 1 being least confident, 10 being most confident), and free-response questions. This was created with the intent to give the residents several different forms to express their feelings on the subject [8]. For question six, specifically “which rheumatology disease do you feel you have the most exposure to?," we kept it open-ended as we did not want to prompt any disease or specify if the exposure was clinically or educationally. The survey was sent out via hospital account email by one of the authors. The residents were all invited to participate and given a 10-day period to respond to the questionnaire. It was sent out on March 20, 2024, and ended on March 30, 2024. The goal was to have the intern class have most of the academic year completed when evaluating their understanding and exposure for accuracy. Participation was voluntary, and the surveys were submitted anonymously, so the authors were blinded to which resident had which response. 

The intent of the survey was to help focus on the weaknesses of the program as felt by residents with the intent to shape future curriculums. The end goal of residency is to create physicians comfortable with treating a wide range of conditions. Within internal medicine, rheumatologic conditions are included in that wide range and creates board-certified physicians. Accordingly, these areas were addressed in the survey. Northwell Institutional Review Board approval was submitted (Ref. No. HSRD HSRD24-0080), and the project was deemed exempt and was considered not human subject research.

## Results

**Figure 1:**
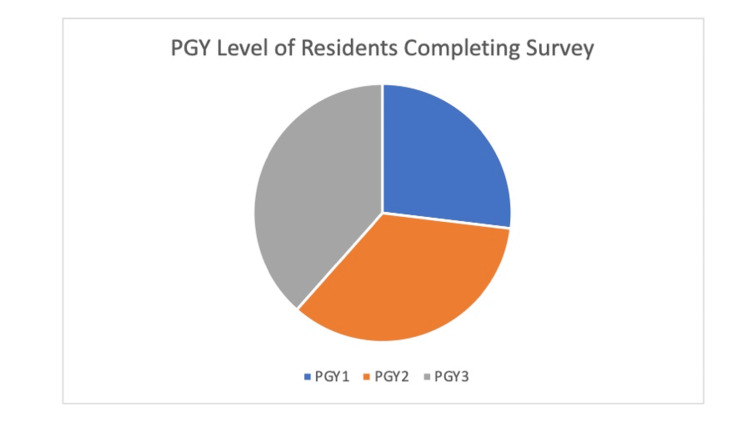
Pie chart depicting the proportions of residents from each class who took the survey. PGY stands for post-graduate year.

**Figure 2:**
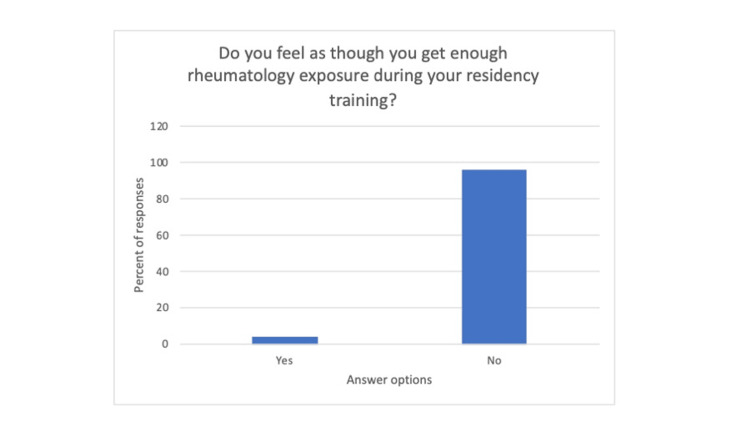
The amount of responses to each numerical option response to the question “What is your confidence in the rheumatology section of your boards?”

**Figure 3:**
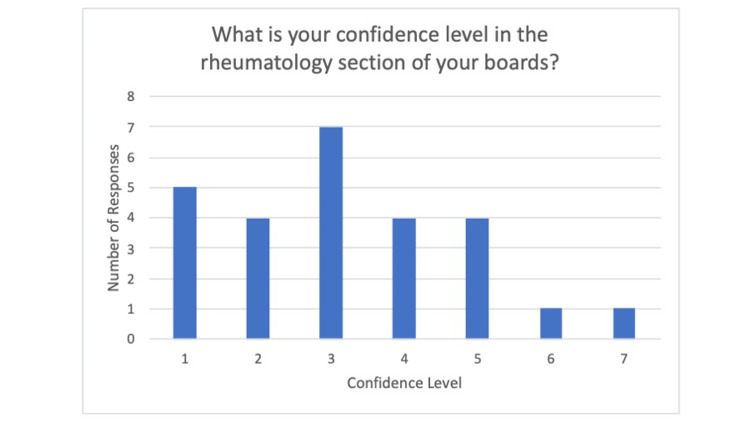
Bar graph depicting percentage of responses stating yes/no to question 2.

The survey was taken by a total of 26 residents. This consisted of 27% PGY1, 35% PGY2, and 38% PGY3 (Figure 1). They were asked five further questions, two multiple-choice, two scaled-voting questions, and one free response. The first question asked “Do you feel as though you get enough rheumatology exposure in your residency training?” (Figure 2) with the answer options of yes or no. To this, 96% of the residents responded no. The next question was “do you feel prepared to treat patients with rheum conditions based on your training?” with the answer options of yes or no. To this, 96% of the residents responded no. The next question asked, “What is your confidence in the rheumatology section of your boards? (1-10, 1 being least confident, 10 being most confident)” (Figure 3). The average of the responses was a three. There was no recorded response above seven. Next, the survey asked, “What is your confidence in conducting an MSK exam? (1-10, 1 being least confident, 10 being most confident).” The average recorded response was a five. The last question asked, “Which rheumatology disease do you feel you have the most exposure to?.” Out of the 23 who utilized the survey 46% responded lupus, 26% responded rheumatoid arthritis and 15% responded saying “none.”

## Discussion

Residents train to become confident in treating patients and to successfully pass their boards at the end of residency. We found an area that needs more attention during training as over 95% of internal medicine residents voiced that they did not get enough exposure and training in rheumatology. This survey spanned all postgraduate classes, had above 25% representation of each, and had a relatively equal distribution. Among all classes, it was shown that they felt there was a lack of preparation and exposure. This shows no correlation with the increase in time of residency and increase in exposure and confidence in the subject. In 2011, a Canadian nationwide survey by Katz concluded that residents had lower confidence in rheumatology than other internal medicine subspecialties, including gastroenterology and cardiology. It was reported that resident confidence improved with teaching as opposed to experience [2]. 

Previous studies have shown a correlation between internal medicine residents' confidence in rheumatology and how their educators perceived their proficiency [9]. This suggests that the knowledge gap is known by preceptors and attendings; however, the initiative for education has not been in effect. Leverenz in particular used in training exams (ITE) scores to help understand resident needs. Sessler has shown that the ITE test is known to not have correlation with resident practice; however, the utility was to identify areas that need more attention in education [4,10]. At their particular institution, they saw that topics that residents had the most confidence in, such as osteoarthritis, were the ones they scored the worst on with their ITE. These data helped us understand the implementation of ITE scores in assessing knowledge. With the gap in confidence and performance, we decided to focus our education curriculum by going independently of previous scores.

Studies were done on the teaching style and preferences of internal medicine residents and rheumatology fellows and residents [11]. It concluded that residents and attendings preferred case-based learning and bedside teaching [12,13]. The American College of Rheumatology has developed “Rheum 2 Learn,” a curriculum to help educate regarding several topics ranging from the musculoskeletal exam to Sjorgens disease. These educational modules are described as “Fundamental Rheumatology Education Designed for Residents.” These consist of cases and quizzes, which help cater to the preference of trainees as stated prior [14]. We now plan to implement and use them for teaching as their availability addresses an area of need. While exploring comparative internal medicine subspecialties (i.e., American College of Gastroenterology and Endocrinology), we saw that their learning was more based on grand rounds and courses as opposed to modules that can be completed on one's own time.

There are several limitations to our study. Our study is limited to a single residency program where the shared opinions of residents could impact the results. The information could be strengthened by expanding to multiple internal medicine programs. Furthermore, our sample size was roughly 33% of the residency program. An increase in the number of residents would help strengthen our data. This study was done at a community hospital with no fellows. Residents communicate directly with attending rheumatologists. Data exploration can aim to compare the confidence and preparation of residents in a program with rheumatology fellows and programs without a fellowship. It is expected that in a hospital with fellows, there is more interdisciplinary communication that can help foster education [15]. However, previous studies done at large academic institutions with fellows have shown similar results. Furthermore, we focused on subjective measurement of resident knowledge and exposure rather than objective data [5]. However, alongside that study, our data continue to show what other similar studies do, as there are multiple data points showing that rheumatology education is lacking in internal medicine residency.

## Conclusions

Over the past 10 years, there have been studies done showing the lack of internal medicine resident confidence in rheumatology. The residents at our program voiced a strong concern for lack of exposure and education. Other studies and institutions have shown this to be a problem that has also been seen with poor testing performance on the subject. While we are not able to identify why there is a lack of attention on increasing knowledge of this field, we believe it is necessary to address going forward.
